# Transcription-induced supercoiling as the driving force of chromatin loop extrusion during formation of TADs in interphase chromosomes

**DOI:** 10.1093/nar/gkx1123

**Published:** 2017-11-13

**Authors:** Dusan Racko, Fabrizio Benedetti, Julien Dorier, Andrzej Stasiak

**Affiliations:** 1 Center for Integrative Genomics, University of Lausanne, 1015-Lausanne, Switzerland; 2 SIB Swiss Institute of Bioinformatics, 1015-Lausanne, Switzerland; 3 Polymer Institute of the Slovak Academy of Sciences, 842 36 Bratislava, Slovakia; 4 Vital-IT, SIB Swiss Institute of Bioinformatics, 1015-Lausanne, Switzerland

## Abstract

Using molecular dynamics simulations, we show here that growing plectonemes resulting from transcription-induced supercoiling have the ability to actively push cohesin rings along chromatin fibres. The pushing direction is such that within each topologically associating domain (TAD) cohesin rings forming handcuffs move from the source of supercoiling, constituted by RNA polymerase with associated DNA topoisomerase TOP1, towards borders of TADs, where supercoiling is released by topoisomerase TOPIIB. Cohesin handcuffs are pushed by continuous flux of supercoiling that is generated by transcription and is then progressively released by action of TOPIIB located at TADs borders. Our model explains what can be the driving force of chromatin loop extrusion and how it can be ensured that loops grow quickly and in a good direction. In addition, the supercoiling-driven loop extrusion mechanism is consistent with earlier explanations proposing why TADs flanked by convergent CTCF binding sites form more stable chromatin loops than TADs flanked by divergent CTCF binding sites. We discuss the role of supercoiling in stimulating enhancer promoter contacts and propose that transcription of eRNA sends the first wave of supercoiling that can activate mRNA transcription in a given TAD.

## INTRODUCTION

In recent years, technological development of Chromosome Conformation Capture (3C) techniques resulted in a rapid succession of ever more illuminating insights into organization of chromosomes in eukaryotic cells (1–7). The majority of these insights focused on the organization of interphase chromosomes, as these partially decondensed chromosomes maintain a complex structure needed for the regulated expression of genes specific for a given tissue as well as of house-keeping genes expressed in all cells (8).

Since 2012 it is known that interphase chromosomes are composed of sequentially arranged, up to megabase-long chromatin domains that show increased frequency of internal contacts (2,3,9). These domains were given the name of topological domains (2) or topologically associating domains (TADs) (3) in a reference to topological domains characterized earlier in bacterial chromosomes (10). On chromosomal contact maps, obtained by such methods as Hi-C (1), the individual TADs manifest themselves as triangles corresponding to regions with locally increased contact frequencies (2). On average, two loci, separated by the same genomic distance contact each other two to three times more frequently when they are located in the same TADs as compared to loci located in two neighbouring TADs (3,8,11). TADs are needed for the regulation of gene expression and particularly to facilitate contacts between enhancers and their target promoters (8,12,13). As a corollary, enhancers and their target promoters are almost always located in the same TAD, even if the genomic distance between them can be as large as a megabase (4,14,15). Natural mutations or genetic manipulations eliminating a border between two neighbouring TADs lead to a misregulation of genes located in the affected TADs (16). Although, it is accepted now that the role of TADs is to facilitate contacts between *cis*-regulatory elements of gene expression located in the same TAD (8), we still do not know what causes the increased frequency of contacts within TADs and how TADs are generated.

Early models proposed that TADs are formed and maintained by polyvalent binders that are specific for individual TADs and which can bridge multiple regions of the same TAD, thus increasing frequency of intra-TAD contact (17). Newer models proposed that TADs are supercoiled and it is supercoiling that increases intra-TAD contacts (11). Currently favoured models of TADs formation involve active process of chromatin loop extrusion (5,18). These models combine earlier models proposed for condensin action during mitotic chromosome condensation (19,20) with more recently established facts that such chromosomal proteins as CTCF and cohesin localize at borders of TADs (2,21,22). Loop extrusion models propose that cohesin rings, presumably in a form of so called cohesin handcuffs (5,23), load on chromatin fibres in such a way that a small chromatin loop passing through the two cohesin rings is formed. These small loops were proposed to increase with time as a result of active translocation of cohesin rings with respect to the chromatin fibre passing through them. The growth of these loops was proposed to stop when translocating cohesin rings reach CTCF proteins at the borders of TADs (5,18). Depending on the orientation of CTCF binding sites, and thus on the imposed orientation of CTCF proteins bound to these sites, with respect to translocating cohesin rings, two different outcomes were proposed. If C terminus of CTCF protein, which is known to bind cohesin (24), is exposed for contacts with approaching cohesin, the formed chromatin loop gets stabilized. If CTCF protein is oriented in the opposite way, its C terminus is unavailable for contacts with cohesin and there is no stabilization of the formed loop as cohesin rings dissociate. Numerical simulations of chromatin loop extrusion produced chromosomal contact maps that matched well the experimental contact maps (5,18). There is however an important potential problem with the chromatin loop extrusion model. It is not known yet what drives the process of the loop extrusion. Cohesins themselves are adenosine triphosphatases (ATPases) and in principle could use the energy gained from ATP hydrolysis to actively translocate along chromatin fibres (18). However, numerous biochemical tests did not reveal yet active chromatin translocation of cohesin rings (25). ATP hydrolysis rates observed in experiments are only compatible with the models where ATP hydrolysis is needed for attaching and detaching of cohesin rings from chromatin fibres (26). Of course, the chromatin loop extrusion model would also work if cohesin rings were pushed by other proteins that actively translocate along the DNA such as RNA polymerases (27). However, RNA polymerases could only push the cohesin rings along the gene body and only in the direction of transcription. It is known though that transcribed portions of genes occupy only a small fraction of TADs length (2) and therefore RNA polymerases would not be able to drive extrusion of chromatin loops larger than individual genes. There are many other motor proteins that can act as DNA translocases (28) and they could push cohesin rings over entire TADs. However, these DNA translocases would need to ‘know’ in which direction they should push the cohesin rings so that the loop growth would end up at TADs borders and would not take too much time. That time should not be longer than several minutes as cohesin rings spontaneously detach from chromatin fibres after a few minutes unless they can form stabilizing interaction with correctly oriented CTCF proteins located at TADs borders (25). Therefore, for a sufficiently rapid loop extrusion its mechanism should be reliable and fast.

We propose here a model of such a mechanism. Our molecular dynamics simulations show that transcription-induced supercoiling acting on chromatin with assembled cohesin handcuffs can drive very efficient chromatin loop extrusion by pushing each of the handcuff-forming cohesin rings towards two opposing borders of the same TAD.

## MATERIALS AND METHODS

### Description of the model

We performed molecular dynamics simulations using Extensible Simulation Package for Research on Soft Matter (ESPResSo) (29). We modified standard beaded chain model to include dihedral potential allowing us to account for torsional tension arising in chromatin fibres. Modifications of beaded chains needed to have the elastic resistance to applied torque were described earlier (30,31). We also accounted for hydrodynamic drag resulting from translational and rotational motion of chromatin fibres in solution. Non-standard features built-in into our models included active swivels that introduce negative supercoiling and thus emulate topological consequences of a combined action of RNA polymerase, producing domains with positive and negative supercoiling, and of TOP1 topoisomerase associated with RNA polymerase in such a way that it can preferentially relax domains with positive supercoiling (32). Modelling of active swivels was as described earlier (30,33). Passive swivels permitting relaxation of torsional stress consisted of sites where the dihedral potential was set to zero, as it was described earlier (33). The phantom sites permitting intersegmental passages and thus mimicking the action of type II topoisomerases (TOPIIB) consisted of short regions of modelled chains, where the excluded volume potential was set to zero. Consecutive main chain beads (M) are bonded by harmonic potential (FENE) with the equilibrium bond length set to 1σ. For modelled chromatin fibres *σ* = 10 nm and corresponds to ca 400 bp. The bending persistence length was set to 50 nm.

The torsional persistence length of chromatin fibres was reported to be as low as 5 nm (34). However, this very low torsional persistence length was only observed in a particular regime where introduced positive supercoiling induced a phase transition between standard nucleosomes, in which their incoming and outgoing linkers were forming negative crossings, and torsionally stressed nucleosomes, in which their incoming and outgoing linkers were forming positive crossings (34). In this regime, especially when the analysed chromatin fibre was in the middle of this transition, the chromatin is torsionally very flexible, with an estimated torsional persistence length of ca 5 nm (34). However, in experiments in which low level of negative supercoiling was introduced to originally unstressed chromatin fibre, the torsional resistance of chromatin fibres to further supercoiling was significantly exceeding this of DNA molecules of the same size (35). Since, we were only interested in the effect of negative supercoiling introduced during transcription, we used the same torsional persistence length as used earlier to model supercoiled DNA molecules (31). To verify whether a particular setting of the torsional persistence length of our model could significantly affect the conclusions of our study, we also performed simulations of chromatin fibres with 10 times lower torsional stiffness than in the simulations presented in the main text. The geometry of formed plectonemes was hardly affected by this decrease of torsional stiffness and most importantly when the plectonemes with lower stiffness were growing, they maintained the ability to push cohesin handcuffs (see Supplementary Movie S1 and Figure S1).

Modelled cohesin handcuffs consisted of two beaded rings joint together and thus forming a figure-of-eight like object. The total size of the cohesin handcuffs is 29 beads, 15 beads per each ring (one bead is common for both rings). Each of cohesin beads has a radius equal to 1σ, corresponding to 10 nm. The external diameter of each of the rings is 45 nm, and the inner diameter is in average 25 nm. Strong and rigid tensile and bending potentials were used to keep the shape of the cohesin handcuff in a planar 8-like structure.

To emulate the rotational and translational drag opposing the movement of chromatin portions passing through cohesin rings, these portions were transiently given large hydrodynamic drag. During ongoing simulations, the coordinates of centres of each cohesin ring were calculated to identify which are the nearest to them beads of the modelled chromatin fibre that was passing through these rings. The hydrodynamic drag of these chromatin beads was increased 10-fold as compared to their state before and after the passage through cohesin rings. Since the motion of tight cohesin rings around and along enclosed chromatin fibres is all the time opposed by intermolecular friction, we accounted for it by increasing two-fold the hydrodynamic drag experienced by all beads forming modelled cohesin rings as compared to beads forming generic chromatin fibres.

## RESULTS

### Modelling of transcription-induced supercoiling

**Figure 1.**
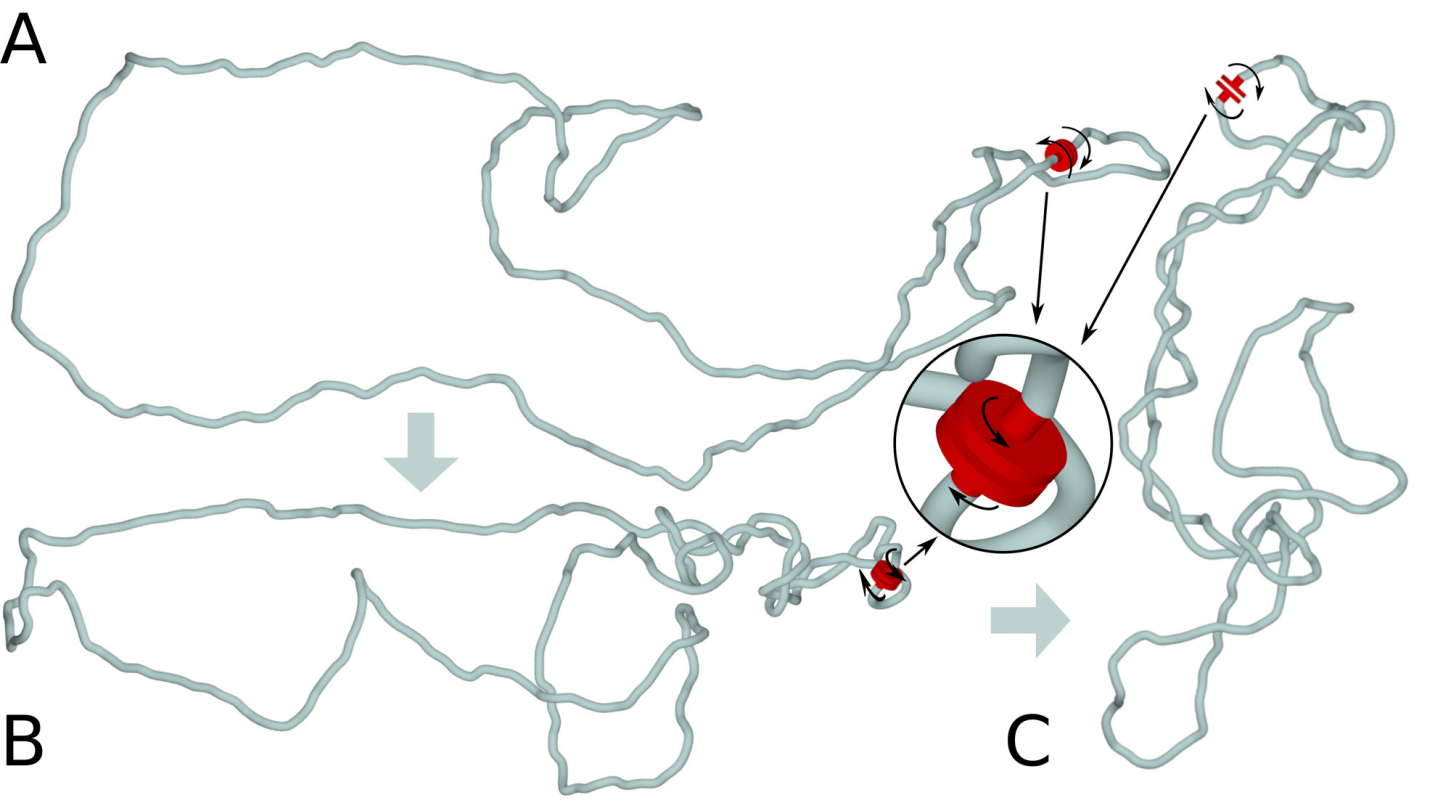
Active swivel that mimics a joint action of RNA polymerase with associated TOP1 introduces negative supercoiling into modelled chromatin fibre. (**A**) A snapshot of starting, thermally equilibrated configuration of a circular, coarse-grained chromatin fibre with the length corresponding to 120 kb and with one active swivel (shown in red). The active swivel is not working yet. (**B** and **C**) Snapshot of configurations obtained after the active swivel performed about 5 and 10 rotations, respectively. The inset shows ideograms of active swivels and indicates their locations. Segments of modelled chromatin fibre that flank the active swivel are forced to swivel with respect to each other. The direction of active swivelling is such that it introduces negative supercoiling, which results in formation of right-handed, interwound plectonemes. In all our figures, to visualize better the structure of plectonemically wound supercoiled regions, the chromatin fibres are shown with the diameter corresponding to 0.3σ instead of 1σ.

**Figure 2.**
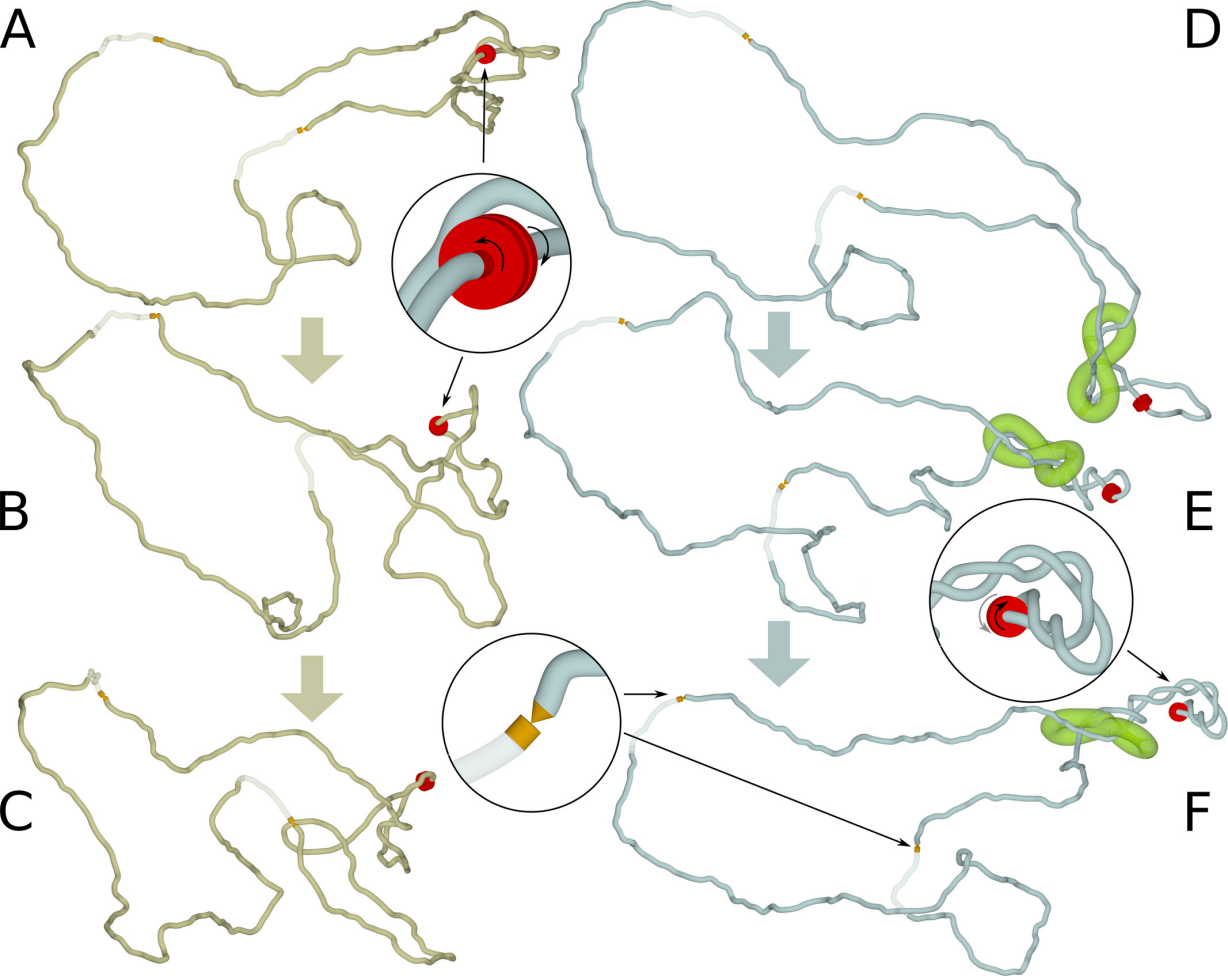
Consequences of limiting axial rotation of chromatin by cohesin handcuffs. (**A**–**C**) Modelling of TADs in which supercoiling can freely diffuse and be dissipated at TADs borders. (A) Starting, equilibrated configuration of modelled TAD with the active swivel ready for action and two sites where supercoiling can be dissipated. (B and C) two snapshots taken after 5 and 10 rotations of the active swivel, respectively. (**D**–**F**) Modelling of TADs in which free diffusion of supercoiling is strongly limited by cohesin handcuffs. (D) Starting, equilibrated configuration of modelled TAD, such as shown in A, but having in addition cohesin handcuffs placed between the active swivel and the TADs borders where supercoiling can be dissipated. (E and F) Two snapshots taken after 5 and 10 rotations of the active swivel, respectively. Notice accumulation of supercoiling in the chromatin portion between active swivels and cohesin handcuffs. Insets show ideograms and locations of active and passive swivels. Passive swivels are presented as sharp conical tips in contact with opposing surface. Torsional stress can freely dissipate by swivelling occurring at passive swivels. As mentioned in the legend to Figure 1, for better visualization of supercoiled regions chromatin fibres are shown with the diameter corresponding to 0.3σ instead of 1σ. However, the modelled cohesin handcuffs are presented with the diameter of 1σ, corresponding to the diameter they had during simulations.

Recent studies of transcription in human cells revealed that type I topoisomerase (TOP1) is physically associated with RNA polymerase (32). This association was proposed to place TOP1 at the front of transcribing polymerase (32). It is well established that during transcription there is formation of ‘twin’ supercoiled domains, with one negative domain generated behind and one positive domain generated ahead of transcribing RNA polymerase (36). Therefore, TOP1 acting ahead of RNA polymerase is ideally positioned to relax positive supercoiling arising ahead of transcribing RNA polymerase but not the negative supercoiling generated behind the polymerase (32). That specific association between RNA polymerase and TOP1 explains why transcribed chromatin domains accumulate net negative supercoiling, why transcribed chromatin domains keep their supercoiling despite being enriched in TOP1 and why both; ongoing transcription and topoisomerases are needed to produce negatively supercoiled domains of chromatin (37). To account for the consequences of TOP1 relaxation of positive but not of negative supercoiling, we model topological consequences of transcription by directly introducing negative supercoiling into chromatin fibres. Supercoiling is introduced by active swivels, where the portions of modelled chromatin fibres abutting the active swivel are forced to undergo axial rotation in the opposing directions (see Figure 1). Figure 1A–C illustrates how in our simulations one active swivel, mimicking the topological consequences of a joint action of one RNA polymerase with associated TOP1, progressively introduces negative supercoiling into a closed loop of chromatin fibre. Torsional stress resulting from negative supercoiling leads to formation of right-handed interwound plectonemes (38). In our simulations, we use a coarse graining approach and 10 nm chromatin fibres are modelled as generic semi-rigid polymer chains with bending and torsional rigidity values in the ranges reported for chromatin fibres (39,40) (see ‘Materials and Methods’ section for more detail). In the situation presented in Figure 1, the negative supercoiling introduced by the active swivel simply accumulates in the closed chromatin loop. This simple model is to explain how supercoiling is introduced in our simulations and it also serves as an introduction to more complex models where supercoiling can be dissipated by action of topoisomerases located at borders of modelled TADs (see Figure 2.)

**Figure 3.**
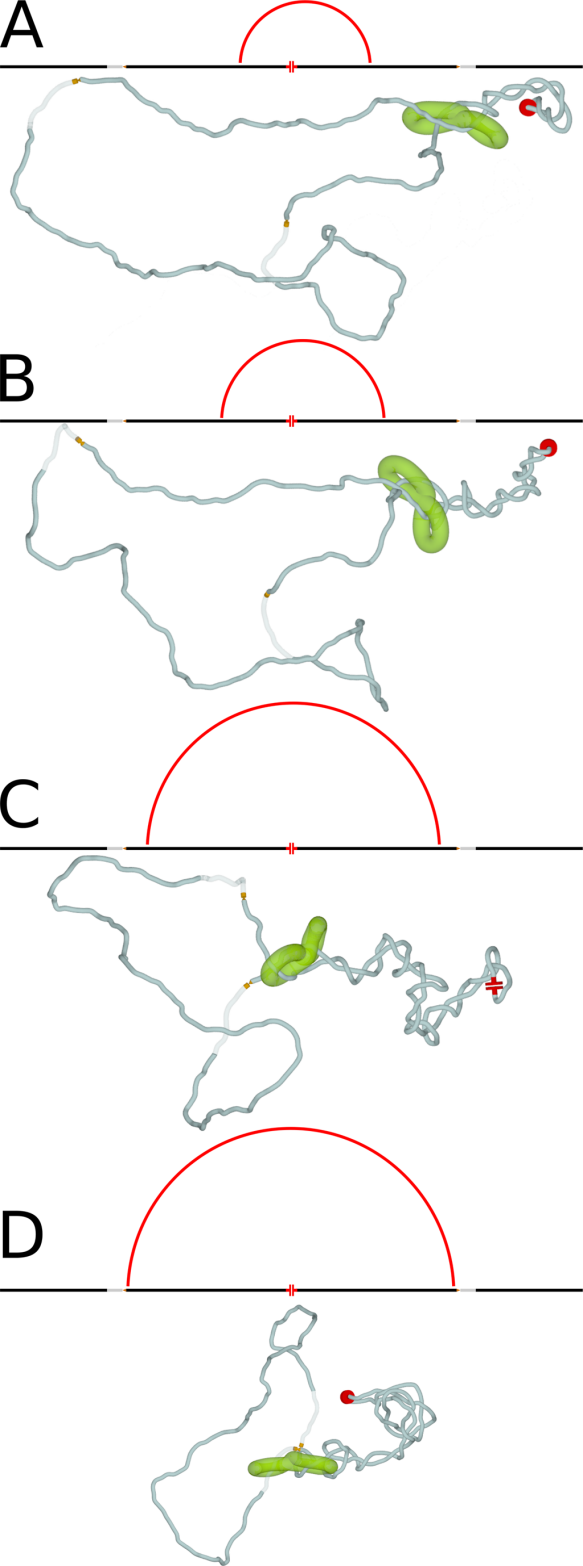
Supercoiling-driven chromatin loop extrusion. (**A**–**D**) Simulation snapshots illustrating how a growing plectonemic region pushes cohesin handcuffs away from the source of supercoiling and towards borders of the modelled TAD. This process eventually brings the two borders of the modelled TAD into close physical proximity (D). Schematic maps, shown above each simulated configuration, illustrate the progress of chromatin loop extrusion process. The loops spanned by cohesin handcuffs grow till reaching the size of the entire TAD. The ends of red arcs correspond to positions of individual cohesin rings forming the handcuff. The linear maps of simulated circular construct are centred at the active swivel. Maps also show position of the two borders of the modelled TAD, with passive swivels and semi-transparent regions accounting for action of type II DNA topoisomerases. Supplementary Movie S2 presents the entire simulation from which the snapshots were taken to compose the figure.

**Figure 4.**
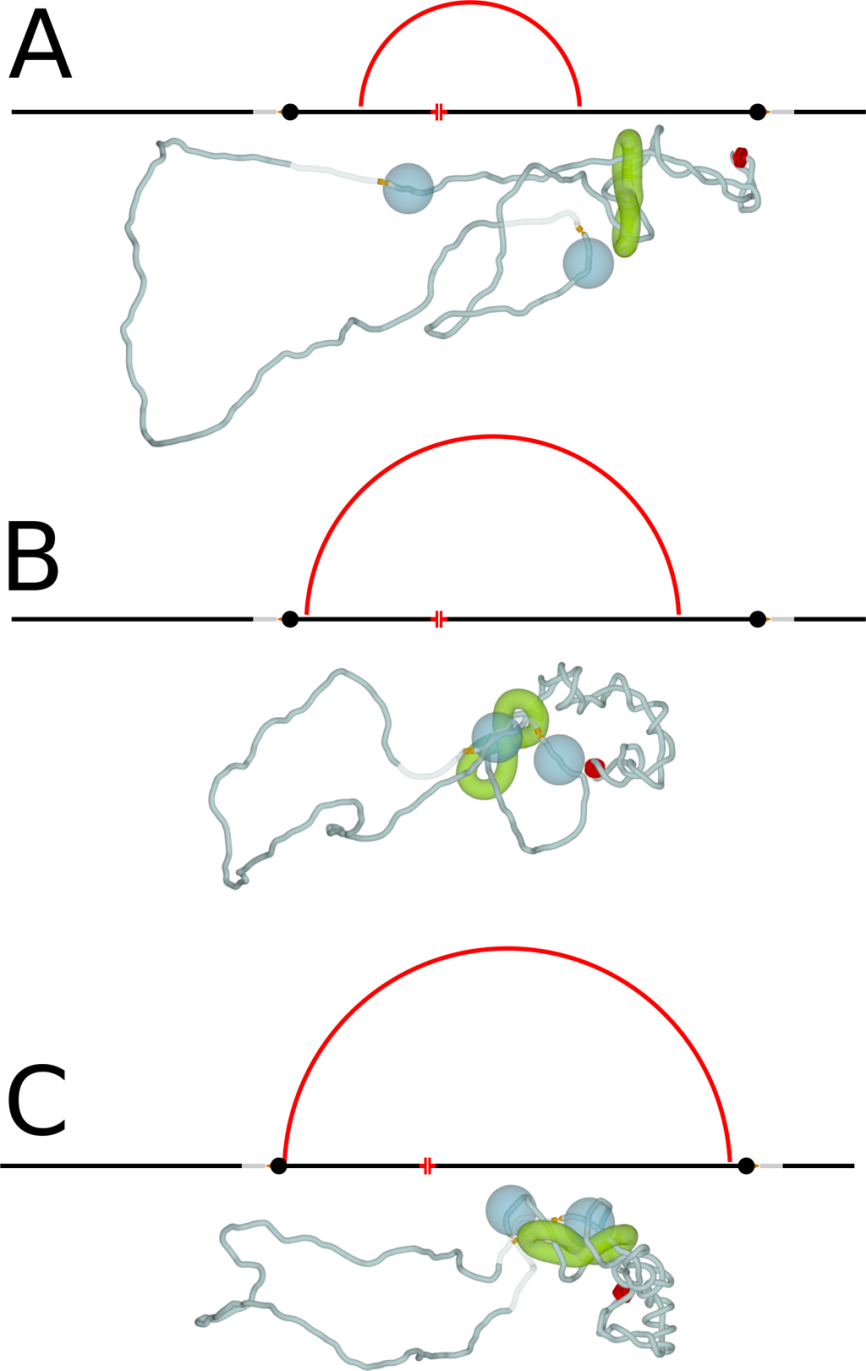
Asymmetric loop extrusion is also efficient in bringing together borders of individual TADs. (**A**–**C**) Simulation snapshots illustrating the progression of supercoiling-induced chromatin loop extrusion when the source of supercoiling is significantly closer to one than to the other border element of the modelled TAD. CTCF proteins bound to CTCF binding sites at borders of TADs are modelled as large beads that cannot pass through cohesin rings. Notice that once one of the cohesin rings forming the handcuff is blocked in its progression by CTCF border element (B) the second ring can still move and eventually reaches the second CTCF border element (C). Therefore, both border elements of modelled TAD, with bound there CTCF proteins can be brought together. As in Figure 3, the schematic maps shown above each snapshot, illustrate the progress of chromatin loop extrusion.

In Figure 1, we presented the final result of accumulation of transcription-induced negative supercoiling which happens when the positive supercoiling is relaxed by TOP1 acting ahead of RNA polymerase. We skipped there several steps needed for the negative supercoiling generated behind transcribing RNA polymerase to find itself on both sides of transcribing RNA polymerase. Transcribing RNA polymerase constitutes a topological barrier restricting diffusion of supercoiling. That topological barrier presumably results from formation of transcription factories grouping several RNA polymerases (41,42). However, once the transcribing RNA polymerase dissociates from the template after terminating transcription, the negative supercoiling accumulated behind RNA polymerase can freely diffuse and redistribute on both sides of the previously transcribed region. Repeated rounds of transcription of a given genomic region permit repeated injections of negative supercoiling. For example, eRNA transcription can provide repeated injections of negative supercoiling. Average size of eRNA is only of about 350 nt (43) but each round of its transcription, taking about 15 s (44), would be sufficient to inject more than 30 negative supercoils into transcribed chromatin fibre (44). After each round of eRNA transcription is terminated, the RNA polymerase dissociation would permit the injected negative supercoiling to spread and equilibrate on both sides of the previously transcribed region (see Figure S2). Since transcribing RNA polymerases bend the DNA and have the preference to localize at apical loops of DNA plectonemes (45), it is natural to expect that transcribing RNA polymerases will also have the tendency to localize at apical loops of chromatin plectonemes. In fact, in our simulations torsional motors mimicking action of RNA polymerase have spontaneous tendency to occupy these positions (see Figures 1–4). A new round of transcriptions occurring in negatively supercoiled chromatin loop makes it in principle possible for the TOP1 positioned ahead of RNA polymerase to relax negative supercoiling that is present in that part of the loop (see Supplementary Figure S2). However, rapid rotation of DNA enforced by transcribing RNA polymerase is expected to create a local wave of positive supercoiling just ahead of transcribing RNA polymerase. Positioning of TOP1 ahead or RNA polymerase makes it likely to relax only positive supercoiling. Therefore, each round of transcription would inject a new portion of negative supercoiling and plectonemes would essentially grow like it is presented in Figures 1–4, although for the sake of simplicity the pulses of transcription and RNA polymerase association/dissociation cycles were not explicitly shown there.

### Cohesin rings constrain axial rotation of chromatin fibres

Although transcription generates negative supercoiling (32), the magnitude of supercoiling does not grow all the time as the transcription progresses (37). The extent of negative supercoiling is controlled, as otherwise DNA helix would become unstable, with such undesirable effects as strand separation or excessive stabilization of R-loops (46–48). The homeostatic mechanisms regulating supercoiling level are well investigated in bacteria (49), but are less known in eukaryotic cells. Recent studies of TADs organization revealed that topoisomerase II beta (TOPIIB) is located right at TADs borders and physically interact with CTCF proteins delimiting individual TADs (50). Type II DNA topoisomerases are the main relaxases of torsional stress in chromatin, as swivelling motion required for action of type I DNA topoisomerases is difficult to achieve in crowded chromatin fibres, whereas there are no such hindrances for topo II-mediated passages between incoming and outgoing linkers of the same nucleosome (51). Therefore, topoisomerases TOPIIB located at TADs borders can act there as torsional stress relaxases, permitting dissipation of excessive torsional stress generated during transcription. To account for the presence of TOPIIB at TADs borders, we introduced additional features into our circular models of individual TADs. Since TOPIIB permit very efficient relaxation of torsional stress in chromatin by a local action involving passages between incoming and outgoing linkers of the same nucleosome (51), we model this type of local action by introducing passive swivels permitting unimpeded swivelling occurring at this site. These swivels are presented in our models as sharp tips touching a flat surface (see the second inset in Figure 2). TOPIIB can also mediate intersegmental chromatin passages between the region where the Topo II is bound and some distal regions. To model this effect, we also introduced phantom-like regions, where modelled chromatin fibres could let other portions of chromatin fibres to pass through. The phantom like regions have no excluded volume potential but their other properties like resistance to stretching, bending or torsional deformation were the same as for the generic parts of the modelled chromatin fibre. The phantom-like regions are shown as semi-transparent regions in Figure 2. Since TOPIIB is known to be located at borders of TADs, we placed swivel sites that were surrounded by phantom sites at both borders of modelled TADs (see Figure 2).

Figure 2A–C shows that when we modelled Topo II localized at TADs borders, there was no accumulation of supercoiling as supercoiling generated in the centre of modelled TAD was dissipating at the sites of topo II action. Efficient relaxation of torsional stress requires that the axial rotation of chromatin fibres is unimpeded so that supercoiling generated at sites of transcription can be transmitted and then dissipated at sites of topo II action. However, the situation changes, when the axial rotation of chromatin induced by transcription is opposed by strong hydrodynamic drag or intermolecular friction (see Figure 2D–F). In such a situation, the diffusion of torsional stress from the site of its generation (RNA Pol +TOP1) to the site of its dissipation (TOPIIB sites) at the border of TAD would be slow and this would lead to formation of plectonemic supercoiling. One of potential biological mechanisms limiting axial rotation of chromatin fibres is likely to involve cohesin rings encircling chromatin fibres. Recent single molecule imaging studies of catalytically active cohesin rings have shown that central pores of cohesin rings are very narrow (25). These studies have shown that cohesin rings encircling DNA are effectively blocked in their diffusion by DNA bound proteins that just slightly exceed the diameter of 10 nm (25). Experiments have shown that already individual nucleosomes assembled on the DNA provide a significant diffusion barrier for cohesin rings sliding along encircled DNA (25). As a corollary, 10 nm chromatin fibre passing through an immobile cohesin ring would be restricted in its ability to rotate.

Studies of Stigler *et al.*, by showing that the central opening of cohesin rings is just large enough to accommodate one 10 nm chromatin fibre, gave also strong support to models proposing that sister chromatids are held together by two cohesin rings in a handcuff-like arrangement (25). Therefore, loop extrusion models involving cohesin should rather operate with cohesin handcuffs than with one cohesin ring embracing two chromatin fibres (25). Figure 2D–F show what happens in a TAD where the axial rotation of chromatin fibres is made difficult by tight cohesin handcuffs. Our modelling shows that negative supercoiling accumulates in the region between the active swivel and cohesin handcuffs (see Figure 2E and F).

The accumulation of supercoiling is the consequence of two factors. Cohesin rings are tight (25) and therefore chromatin fibers are prevented from free rotation within them. However, if individual cohesin rings were not tethered to each other, they could rotate together with enclosed chromatin fibres and this would not restrict axial rotation and dissipation of supercoiling by chromatin fibres at borders of TADs. The handcuff form of cohesin (schematically presented as a figure of eight structure in Figure 2) prevents individual cohesin rings from rotating with respect to each other. If such a rotation is blocked the axial rotations of enclosed chromatin fibres can be strongly limited. Under such conditions plectonemic supercoiling will accumulate in a chromatin portion that contains the source of supercoiling, i.e. in a region with ongoing transcription that is flanked by two cohesin rings forming the same handcuff (see Figure 2E and F). The actual geometry of cohesin handcuffs, including the distance at which two chromatin fibres are maintained by the handcuffs does not seem to be important. Our earlier study showed that growing plectonemes in simulated DNA molecules have the ability to push intervening objects hindering the plectoneme growth (30). For this to occur, there was no need to impose any particular geometry in the region where modelled elastic filaments were approaching each other forming a plectoneme. As there were no cohesin handcuffs separating intertwining filaments, they were free to adopt the geometry minimizing the elastic deformations and this was sufficient to efficiently push away objects interfering with the growth of plectonemes (30).

### Cohesin handcuffs are actively pushed by the accumulated supercoiling towards sites where the torsional stress can be dissipated

Figure 3A–D and supplementary movie (Supplementary Movie S2) show further evolution of the situation presented in Figure 2E and F. The growing plectoneme, resulting from transcription-induced supercoiling, progressively pushes cohesin handcuffs towards TADs borders. As mentioned earlier, cohesin rings embrace quite tightly chromatin fibres (25). Therefore, the motion of cohesin rings around and also along encircled chromatin fibres is opposed by a drag (25). However, when a persistent force pushes cohesin rings along chromatin fibres, the rings can move in the direction dictated by the force. With transcription-induced negative supercoiling generated in the chromatin portion enclosed by cohesin handcuffs and with supercoiling dissipation occurring at TADs borders, the plectonemic region grows and this pushes cohesin handcuffs away from the source of supercoiling towards TADs borders (see Figure 3A–D and Supplementary Movie S2). When cohesin handcuffs are progressively pushed towards TADs borders, the chromatin loops spanned by cohesin handcuffs grow and the entire process is formally similar to proposed earlier chromatin loop extrusion models implicating cohesin rings (5,18). The essential difference, though, with respect to these previously proposed models is the driving force of the loop extrusion process. This driving force in our model is provided by supercoiling generated during transcription.

As already mentioned, we favour the mechanism in which repeated rounds of transcription, followed by RNA polymerase dissociation, permit diffusion of supercoiling through the region that was previously transcribed (see Supplementary Figure S2). However, it is interesting to consider what will happen when the transcription process is non-interrupted for a long time and therefore the generated negative supercoils cannot diffuse through topological barrier consisting of transcribing RNA polymerase. Our simulations of such a situation have shown (see Supplementary Figure S3 and Movie S3) that plectonemes still form but they are composed of one chromatin fibre that is torsionally relaxed and the second one that is negatively supercoiled. The torsionally relaxed fibre takes the axial position in the plectoneme, whereas the negatively supercoiled fibre wounds in a right-handed way around the torsionally relaxed central fibre (See Supplementary Movie S3). Importantly, as negative supercoiling accumulates these hemi-supercoiled plectonemes grow and they push the modelled cohesin handcuffs in a similar way as it was observed in standard, negatively supercoiled plectonemes presented in Figure 3. Interestingly, the TOP1 acting ahead of RNA polymerase is unlikely to relax these hemi-supercoiled plectonemes as TOP1 position makes it likely to act and relax only the chromatin fibre that is already torsionally relaxed (See Supplementary Figure S3 and Movie S3). Since all our results can be achieved with standard negatively supercoiled plectonemes, we only mention here the possibility of forming dynamic hemi-supercoiled plectonemes and we will explore their possible role in our future studies.

### Supercoiling-driven chromatin loop extrusion may proceed asymmetrically

**Figure 5.**
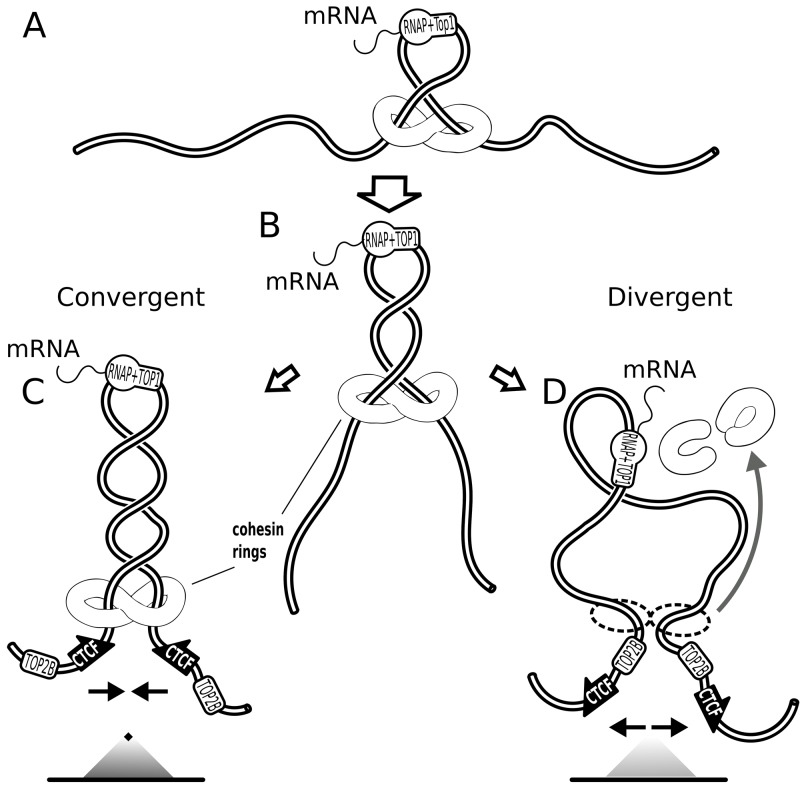
Pushing of cohesin handcuffs by supercoiling can also explain why the orientation of CTCF binding sites determines the stability of chromatin loops forming TADs. (**A**) Transcribing RNA polymerase in association with TOP1 induces formation of negative supercoils. Cohesin handcuffs load near a crossing caused by negative supercoiling. (**B**) The growing plectoneme pushes cohesin handcuffs irrespectively of the orientation of CTCF sites at TADs borders. (**C**) When CTCF binding sites are convergent, the C terminal parts of bound CTCF protein can contact cohesin rings and this interaction stabilizes cohesin handcuffs and formed chromatin loops. On contact maps, such TADs form triangles with strong tips. (**D**) When CTCF binding sites are divergent, the C terminal part of bound CTCF protein is unavailable for contacts with cohesin rings. Without these stabilizing interactions cohesin rings dissociate from chromatin and this permits rapid relaxation of accumulated torsional stress by TOP2B that is normally associated with the N terminal part of bound CTCF. On contact maps, such TADs form triangles without strong tips.

In simulations shown in Figures 2 and 3 the active swivel producing negative supercoiling, and thus mimicking topological effects of ongoing transcription, was placed in the region that was equally distant from the two borders of the modelled TAD. However, transcribed genes are unlikely to be placed exactly in the middle of topological domains in which they reside. Therefore, we decided to test by simulations what is likely to happen when the source of supercoiling is located asymmetrically within a TAD. In addition, we assembled cohesin handcuffs so that in the initially formed loop one of cohesin rings forming the handcuff was significantly closer to the active swivel than the other cohesin ring. To make our simulated system closer to what is known about TADs, we accounted for the known fact that CTCF proteins are located at borders of TADs. We also took into account that CTCF proteins are frequently bound by other proteins (52) resulting in a bulky obstacle that is likely to be too large to pass through cohesin rings (25). We therefore modelled CTCF proteins, bound at TADs borders, as large beads that are bigger than openings of modelled cohesin rings. Figure 4A–C shows what happens when we model such a situation. As in the symmetric situation presented in Figure 3, supercoiling accumulates in the chromatin portion containing the source of supercoiling and cohesin handcuffs limiting diffusion and dissipation of supercoiling (see Figure 4A). Once the plectoneme forms, both cohesin rings in a handcuff are pushed with a similar speed by the growing supercoiled region and this continues till one of the cohesin rings reaches the bulky CTCF protein (Figure 4B). From that point on, one of the cohesin rings stops its progression. However, as the supercoiling is still generated and as the formation of longer plectonemically wound regions decreases the elastic energy of modelled elastic filaments, an interesting slithering motion is observed. That motion causes progressive phase shift within supercoiled region, which permits formation of a longer plectoneme and brings together two borders of the modelled TAD (see Figure 4C). Therefore, even if the transcribed gene is located peripherally in a TAD, the generated supercoiling can still bring together the border elements of that TAD. The only requirement would be that cohesin handcuffs flank the source of supercoiling i.e. the transcribed portion of chromatin within a given TAD. Although, it is known that transcription-induced supercoiling is generated more efficiently in regions with converging or diverging directions of transcription (53,54), our model is not dependent on any particular gene arrangement. In addition, supercoiled loops generated by individual transcribing polymerases naturally provide correctly positioned loading sites for cohesin handcuffs (see Figures 4 and 5) suggesting that loading of cohesin handcuffs on chromatin loops may also be regulated by supercoiling.

## DISCUSSION

We showed here how transcription-induced supercoiling can actively push cohesin rings and thus drive chromatin loop extrusion implicated in TADs formation. The important difference between this novel model and earlier models of chromatin loop extrusion is that in our model the translocation of cohesin rings is driven by transcription induced supercoiling, which is a well-documented process occurring in living cells (36,37,55–57). The earlier models assumed that cohesin rings are themselves active DNA translocases (5,18). However, there is no experimental evidence yet supporting active translocation of cohesin rings (25). Even if cohesin rings were able to actively translocate along the DNA, there is a problem how they could ‘know’ which way they should move along chromatin fibres to reach CTCF protein bound at TADs borders. The same problem with the direction of movement would apply if other translocases were involved in pushing cohesin rings. Our model proposes that transcription-induced supercoiling is not only the driving force for the chromatin loop extrusion but also explains how this driving force is directed from the source of supercoiling (regions of transcriptions) towards TADs’ borders where supercoiling is dissipated by the action of specialized topoisomerases.

Recent ChipSeq experiments have shown that TADs’ borders are indeed the places where TOP2B is bound (50) and thus the places where transcription-induced supercoiling generated in a given TAD can be dissipated. Therefore, in individual TADs there is a flux of supercoiling starting from sites of transcription and ending at TADs borders. Recent single molecule studies indicated that inner diameter of cohesin rings are too small to enclose two chromatin fibres (25). These findings strongly support the handcuff model of cohesin interaction with chromatin (23). In addition, the small inner diameter of cohesin rings results in a significant intermolecular friction between cohesin rings and enclosed chromatin fibres even if just one chromatin fibre passes through a cohesin ring (25).

Our model takes into account numerous studies demonstrating that transcription generates net negative supercoiling (37,56,58). This is the expected consequence of the fact that human TOP1 is associated with the leading edge of transcription machinery and relaxes positive supercoiling generated ahead of transcribing polymerase but not the negative supercoiling generated behind the RNA polymerase (32).

Entering these new experimental findings into our simulated systems, we observed that once cohesin handcuffs are associated with chromatin fibre in such a way that they flank the transcribed regions, the negative supercoiling generated during transcription starts to accumulate in the chromatin loop spanned by the handcuffs (see Figures 2–5) Growing, plectonemically wound region pushes then cohesin handcuff away from the source of supercoiling i.e. towards the two borders of the implicated TADs (see Figures 2–5). Although the two cohesin rings forming a handcuff move together in the same physical direction, they in fact move in opposite direction with respect to genetic map of a given chromosome and therefore the chromatin loop spanned by the handcuffs grows. Our simulations revealed that tight cohesin rings forming handcuffs constitute together an interesting device that can harness the energy of supercoiling to drive the translocation of cohesin rings towards border elements of individual TADs.

With respect to the experimental observation that only TADs flanked by convergent CTCF sites form loops (5,21), our model shows no difference with earlier models. In convergent orientation of non-palindromic CTCF binding sites flanking a given TAD, CTCF proteins are bound to CTCF sites in such orientation that they can contact approaching cohesin with its C terminus, which is known to bind cohesin (24). This interaction leads then to stabilization of cohesin handcuffs on chromatin fibre and this extends the lifetime of formed chromatin loops. As the consequence, the contact map of such a TAD looks like a triangle with a strong tip (see Figure 5C). This is not the case, however, for the other orientation of CTCF binding sites and thus of bound CTCF protein with respect to cohesin handcuffs pushed by growing plectonemes (see Figure 5). In the absence of stabilizing interactions with C terminus of CTCF, cohesin handcuffs pressed against TOP2B protein, which is bound to N terminus of CTCF (50), will be likely to dissociate (see Figure 5D). In such a case, the corresponding TAD will produce on the contact map a triangle without a strong tip (see Figure 5D). In addition, such features on the experimental chromosomal contact maps as lines of contacts where one of the border elements is seen as having increased contact frequency with large portion of the TAD, can be explained by our models in a similar way as by the earlier models (18). These lines of contacts were proposed to arise when one end of the extruded loop already stopped at the TAD border but the other end was still moving (see Figure 4). This happens when the loop extrusion is initiated close to one of the borders of a given TAD. Formation of such contact maps is not expected to depend on the specific mechanism by which the cohesin rings are pushed but on the localization of chromatin region where cohesin rings initially bind to chromatin.

It is interesting to consider how our model is affected by the known fact that there are many weak CTCF sites within TADs. Weak internal CTCF sites, as detected by ChIP-sec methods, can indicate that only a fraction of cells have CTCF strongly bound there or that CTCF is bound to all these sites very weakly. In the first case, cohesin rings would pass through unoccupied sites and would be stopped and then stabilized or destabilized at sites occupied by CTCF. In the second case, cohesin rings may displace weakly bound CTCF proteins and progress further with the loop extrusion or may be stopped at these sites. The probability of displacing CTCF would decrease with the strength of these CTCF sites. In both cases the resulting contact maps would reflect experimental data showing that weak CTCF sites form loops less frequently than strong sites (5). It is important to add here that formed loops are not permanent even if they are formed between convergent CTCF sites (5,18).

Very recent studies showed that when cohesin rings are prevented from loading on chromatin there are practically no TADs visible in affected chromosomes (59). These recent studies strongly supported the chromatin loop extrusion model implicating cohesin rings. However, this new study gives the same support to all models that invoke cohesin rings in formation of TADs, such as the one proposed here or earlier models of chromatin loop extrusion.

**Figure 6.**
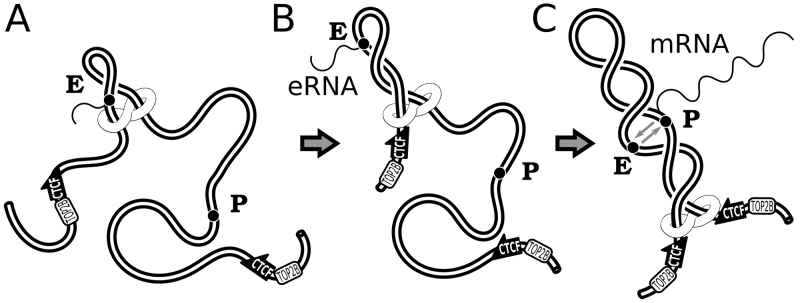
Transcription of enhancers provides the first wave of supercoiling needed for chromatin loop extrusion forming TADs, in which promoters of developmentally regulated genes can contact their partner enhancers. (**A**) Transcription of an enhancer is initiated by the presence of some transcription factor but is independent of contacts with another enhancer. Cohesin handcuffs load at one of the crossings resulting from supercoiling generated during eRNA transcription. (**B**) The growing plectoneme pushes cohesin handcuffs towards borders of the TAD with CTCF binding sites in a convergent orientation at its border. (**C**) Once the growing chromatin loop spans the entire TAD the promoter of developmentally regulated gene can interact with its partner enhancer and start transcription.

We argue here that transcription induced supercoiling is responsible for driving chromatin loop extrusion and thus for shaping TADs in chromosomes of higher eukaryotes. We have proposed earlier that supercoiling of chromatin loops forming TADs is a natural way to increase frequency of intra TADs contacts needed to promote enhancer–promoter interaction (60). This proposition was inspired by earlier studies of bacterial systems (61). Since enhancers and their target promoters reside in the same TAD, it is generally accepted now that the increased frequency of intra-TADs contacts plays a crucial role in the initiation of transcription of developmentally regulated genes (8,12,13). However, if the initiation of transcription of enhancer-dependent genes requires prior supercoiling this prior supercoiling cannot be generated by the transcription of enhancer dependent genes. What can be then the source of transcription-induced supercoiling in TADs? Since 2010 it is known that enhancers are themselves transcribed and their transcripts are known as eRNA (62,63). It is also known that transcription of enhancers precedes the transcription of genes they regulate (64,65). Therefore, transcription of enhancers is likely to introduce the initial wave of supercoiling needed for TADs formation (see Figure 6). Supercoiling generated by the transcription of enhancers can push then cohesin handcuffs and cause chromatin loop extrusion (as we observed it here in our modelled system). Once a given TAD forms supercoiled loop, the increased frequency of intra-TAD contacts can bring together enhancers, whose transcription may not be needed at this point, with their target promoters and the transcription of enhancer-regulated genes can start (see Figure 6). The sequence of events where eRNA transcription is followed by loop formation, which in turn is followed by mRNA transcription of enhancer-regulated genes agrees with sequence of events experimentally observed in β-globin locus (65,66). Our model presented in Figure 6 provides an explanation why transcription of enhancers is needed for action of many enhancer despite the fact that eRNA is frequently quickly degraded (66).

Our simulation studies assumed that negative supercoiling generated during transcription can be long-lived and can spread over long distances. This assumption is based on several studies showing long-lived negative supercoiling maintained over large portions of interphase chromosomes in eukaryotic cells (37,55). However, studies by Kouzine *et al.* (67) propagated the message that transcription can maintain negative supercoiling only over a distance of about 1.5 kb ahead of transcription start sites (TSS). We believe, though, that this 1.5 kb distance refers only to the size of regions with high magnitude of negative supercoiling. Figure 3 of the same paper clearly shows that even at distances of 5 kb ahead of TSS of active genes, negative supercoiling is still present and its magnitude amounts to ca 50% of what is measured just upstream of TSS in these genes (67). Most likely, negative supercoiling spreads much further as there are no known specific topological barriers located ca 5 kb ahead of TSS of active genes. However, Kouzine *et al.* (67) did not report experiments aimed to detect negative supercoiling at larger distances ahead of TSS. The observed progressive decrease of the supercoiling level with the distance from TSS is consistent with our model. If in individual non-synchronized cells plectonemically wound regions grow starting from active genes (as proposed in our model), then a larger fraction of cells will have plectonemes organized near the active gene than further away. Upon averaging over all cells in a sample, this will produce a gradient of supercoiling as it was the case in the study by Kouzine *et al.* (67). More recently, Kouzine *et al.* proposed that transcription of eRNA may have global effects on TADs through generated supercoiling (68), thus recognizing the possibility of long-range action of transcription-induced negative supercoiling.

It is important to add here that our model, which proposes that cohesin handcuffs are pushed by growing plectonemes, is not in contradiction with structural transitions of DNA that are induced by negative supercoiling such as formation of left-handed Z-DNA structure (69). Studies in Levens group revealed that short DNA regions with sequences prone to adopt Z-DNA structure actually undergo these transitions in transcriptionally active cells and such regions are enriched upstream of transcriptionally active genes (70). Structural transition from B- to Z-DNA structure has the capacity to decrease the level of negative supercoiling as roughly two supercoils can be compensated by 10bp switching from right handed B-DNA structure to left-handed Z-DNA (69). However, this capacity is quickly exhausted as Z-DNA forming regions are short and rare. Since RNA polymerase with TOP1 positioned ahead of it, generates 1 negative supercoil per each 10 transcribed bases, the B-Z transitions may at best slow down the accumulation of negative supercoiling. Once the Z-DNA region is formed, there is no reason to expect that a stretch of Z-DNA would pass through cohesin rings differently than a stretch of B-DNA. According to our model, the presence of cohesin rings slows down the dissipation of negative supercoiling at TADs borders and thus increases supercoiling level in TADs. This may explain why transcription-induced transitions to non-B DNA structures, such as Z-DNA, were not limited to regions located just upstream of the active genes (70).

One implication of our model is that supercoiling is expected to be correlated with the position of actively transcribed genes. This agrees with results of Naughton *et al.* (37) showing a strong relationship between negatively supercoiling domains and transcription. However, in the same article, Naughton *et al.* also showed that only about 1/10 of supercoiling domains borders corresponded to TADs borders (37). That latter result was later interpreted by Fudenberg *et al.* (18) as contradicting the idea that supercoiling could explain TADs formation. It needs to be clarified here though that supercoiling domains determined by Naughton *et al.* (37) had an average size of 100 kb and the positions of their borders were compared to TADs borders, determined on low resolution Hi-C maps available at the time. According to those maps the average size of TADs was of about 1 Mbp (2). Therefore, at most 1 per 10 borders of supercoiling domains could have corresponded to these TADs borders. The definition of TADs used in the comparison with supercoiled domains is open to discussion though, as more recent studies report TADs with average size of 180 kb (21). Possibly, with further refinements of TADs maps their position may coincide with positions of supercoiling domains.

There are more studies indicating the presence of negative supercoiling in chromosomes. Yeast chromosomes, which are much smaller than human chromosomes, release accumulated torsional stress by rotation of their ends (58). Psoralen photo-crosslinking studies of yeast chromosome showed a gradient of psoralen binding, which indicates that the interior of yeast chromosomes is negatively supercoiled and the magnitude of negative supercoiling decreases as one approaches telomeres (58).

The model proposed here assumes that negative supercoiling can be long-lived in eukaryotic cells and we discussed earlier the experimental data supporting this assumption. This contrasts, though, with a popular notion that DNA topoisomerases are omnipresent within cells and therefore can quickly relax any torsional stress. However, this popular notion is probably wrong as DNA topoisomerases are highly controlled and regulated in living cells (71). Bacterial cells, for example, keep their DNA negatively supercoiled despite presence of various TOP1 and TOPIIB, which in principle could relax DNA supercoiling. Mechanisms that protect negatively supercoiled DNA from relaxation are in part known. For example, topoisomerase IV that is highly active in relaxing torsional stress in positively supercoiled DNA is very inefficient in relaxing negatively supercoiled DNA (72,73). It has been proposed that differences in the geometry between superhelices turning in left- or right-handed direction strongly affect topoisomerase IV action (73–75). Discovery that chromosomes of higher eukaryotes form chromosomal territories, which do not intermingle with each other (76), revealed that there are essentially no passages between intact chromatin fibres (77). Also Hi-C studies determining the rate with which contacts between chromosomal regions decay as the genomic distance separating these regions increases, revealed that this decay rate is best explained by the inability of DNA topoisomerases to mediate unrestricted passages between chromatin fibres (1). We only recently started to understand that DNA topoisomerases in higher eukaryotes are prevented from uncontrolled activity by binding and direct interactions with other proteins such as discussed earlier interactions with RNA polymerase (32) or CTCF protein (50), but also including specific interactions with chromatin remodellers SMARCA4 and BAF250a (78,79).

Our model postulates that transcription is needed for the formation of TADs. Indeed, recent studies of chromatin structure in inactive X chromosomes showed that a few genes that were transcriptionally active in these chromosomes, were all located in chromosome portions forming TADs, whereas the rest of the chromosome was TADs free (80). However, once TADs are formed and chromatin loops are extruded and stabilized by interaction between cohesin and CTCF, the ongoing transcription would not be necessary anymore for TADs maintenance. Therefore, the observation of Palstra *et al.*, that long-range chromosomal contacts detected by 4C method can be still detected after transcription has been stopped (81), does not speak against our model.

According to our model, relaxation of positive supercoiling by TOP1 is required for the generation of negative supercoiling. This requirement agrees with earlier experiments by Naughton *et al.* (37), showing that inhibition of TOP1 abrogated formation of negatively supercoiled domains in interphase chromosomes.

Our model requires that chromatin fibres passing through cohesin ring experience significant hydrodynamic drag limiting their free rotation. Indeed, earlier experiments by Stiegler *et al.*, observed and measured such a drag (25). That drag was so strong that Stiegler *et al*, calculated that a cohesin ring would need 1 h to diffuse over the distance of 7 kb (25). This speed of diffusion may seem to be much too slow to permit cohesin rings to successfully complete chromatin loop extrusion, during which it would need to traverse several hundreds kb and do it within ca 10 min, which is a typical time of cohesin rings stability on chromatin fibres (25). However, Stiegler *et al.*, calculations were based on observations of thermally driven random walk of cohesin rings enclosing chromatin fibres (25). If cohesin rings are actively pushed in one direction, as in our model, they may move as quick as proposed for active loop extrusion models (5,18).

When our manuscript was under review, new studies were published that demonstrated the presence of active TOP2B at borders of loop-forming TADs (82). The analysis of sites and frequency of observed double strand DNA breaks led the authors to suggest that: ‘DNA extrusion and topological stress relief go hand in hand’. This suggestion agrees with what happens in the proposed here model where supercoiling drives chromatin loop extrusion.

## Supplementary Material

Supplementary Data
