# Supplementary material for: Transcription-induced supercoiling as the driving force of chromatin loop extrusion during formation of TADs in interphase chromosomes
Source: Nucleic Acids Res. 2017 Nov 13;46(4):1648–60. doi: 10.1093/nar/gkx1123 (PMC5829651; doi:10.1093/nar/gkx1123)
Supplement: Supplementary Data [file gkx1123_supp.zip › Captions-to-SI-figures.pdf]

Legends to supplementary figures and movies.

Figure S1.
Cohesin handcuffs are pushed by supercoiling independently of a particular value of torsional stiffness of chromatin fibres.
A-C. Simulation snapshots showing the progress of chromatin loop extrusion when the torsional stiffness of modelled chromatin fibres was decreased 10-fold, as compared to simulations presented in the main text.

Figure S2.
Cycles of RNA polymerase transcription and dissociation are needed for the diffusion of negative supercoiling over previously transcribed region.
A-B. When TOP1 relaxes positive supercoiling generated ahead of transcribing RNA polymerase there is an accumulation of negative supercoiling behind transcribing RNA polymerase. C. Once RNA polymerase associated with TOP1 finishes transcription and dissociates from the template, the negative supercoiling accumulated behind the RNA polymerase can diffuse and spread over previously transcribed region. D-E. A new round of transcription injects negative supercoiling behind transcribed region. F. Spreading of supercoiling over previously transcribed region.

Figure S3.
Hemi-supercoiled plectonemes have the ability to drive chromatin loop extrusion.
A-C. Simulation snapshots showing progress of chromatin loop extrusion under conditions where negative supercoiling generated behind transcribing RNA polymerase is prevented from diffusing over the transcribed region. To mimic this condition negative supercoiling was introduced only on one side of the active swivel, whereas the other side was prevented from any rotation. Interestingly, plectonemes still form but have a different structure. Non-supercoiled fibre takes the central position in the plectoneme, whereas supercoiled fibre winds in a right handed way around the non-supercoiled fibre. As supercoiling is generated, the hemisupercoiled plectoneme grows and pushes cohesin rings.

Movie S1.
Simulation run testing effects of decreased torsional stiffness of chromatin fibres on supercoiling-driven loop extrusion.

Movie S2.
Simulation run illustrating how cohesin handcuffs are pushed by growing negatively supercoiled plectonemes.

Movie S3.
Simulation run testing what happens when negative supercoiling generated behind transcribing RNA polymerase is prevented from diffusing over the transcribed region.
