# Supplementary figures and images for: Transcription-induced supercoiling as the driving force of chromatin loop extrusion during formation of TADs in interphase chromosomes
Source: Nucleic Acids Res. 2017 Nov 13;46(4):1648–60. doi: 10.1093/nar/gkx1123 (PMC5829651; doi:10.1093/nar/gkx1123)

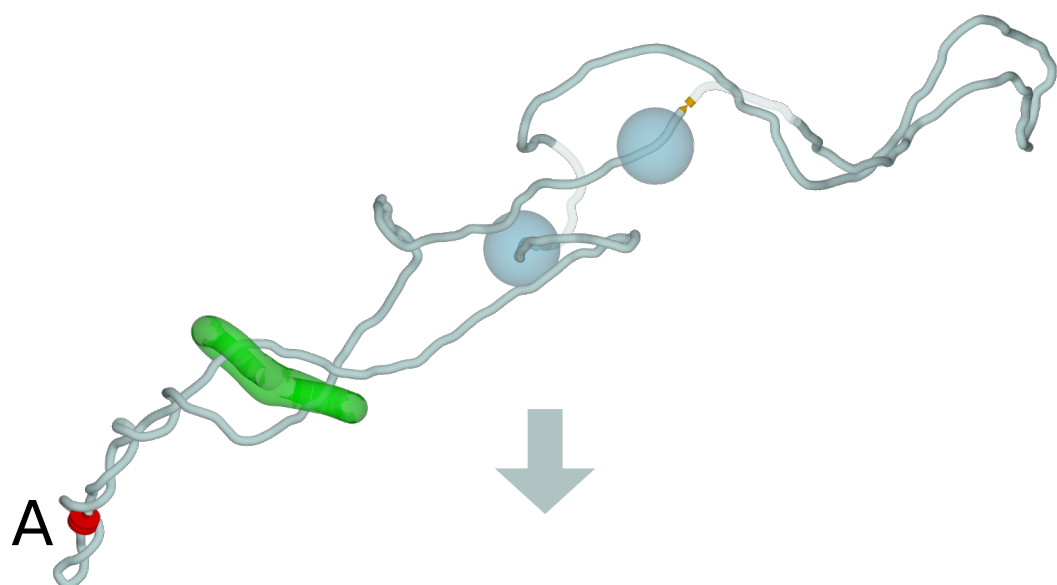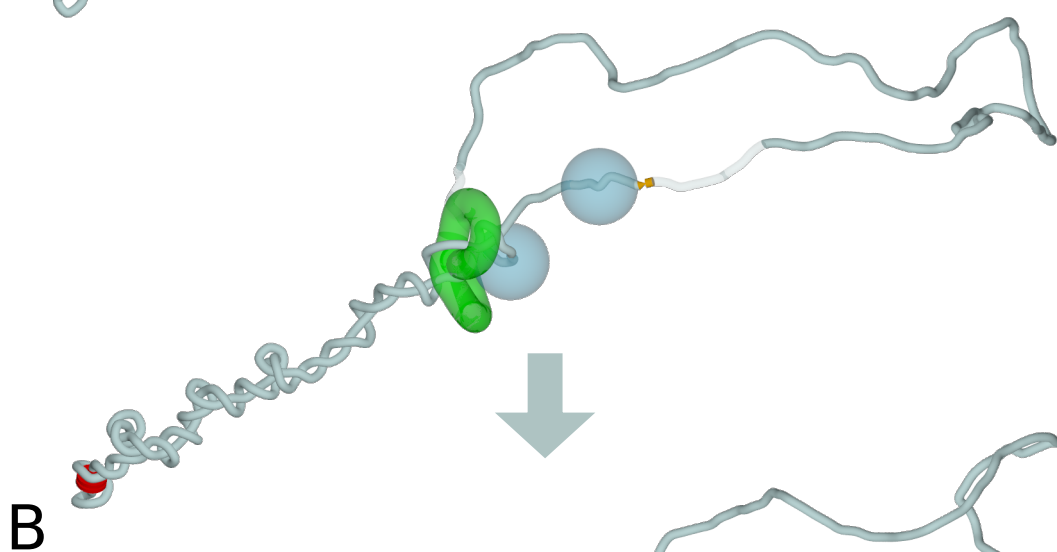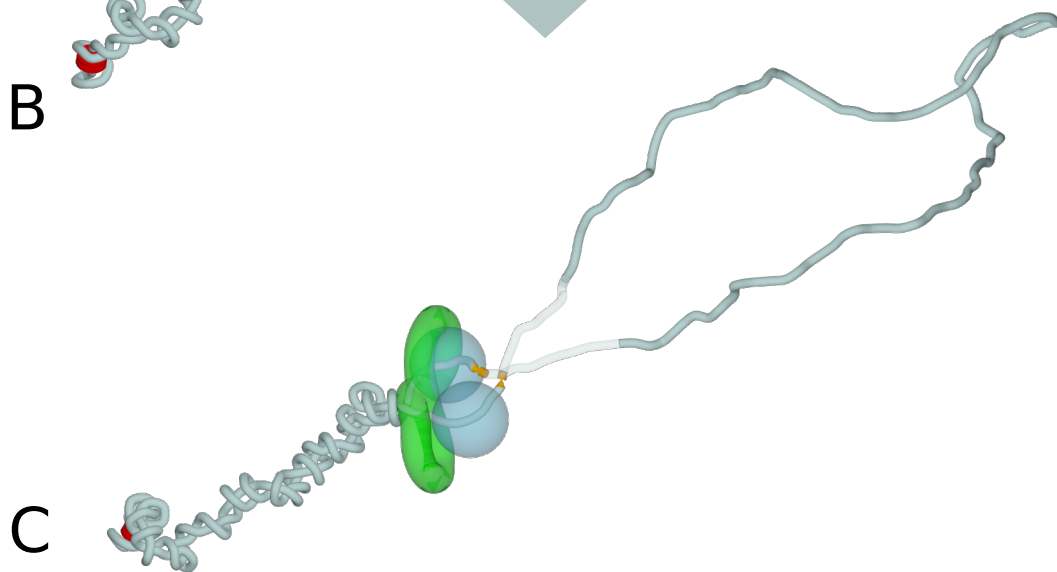

Supplement: Supplementary Data [file gkx1123_supp.zip › Figure S2.pdf]

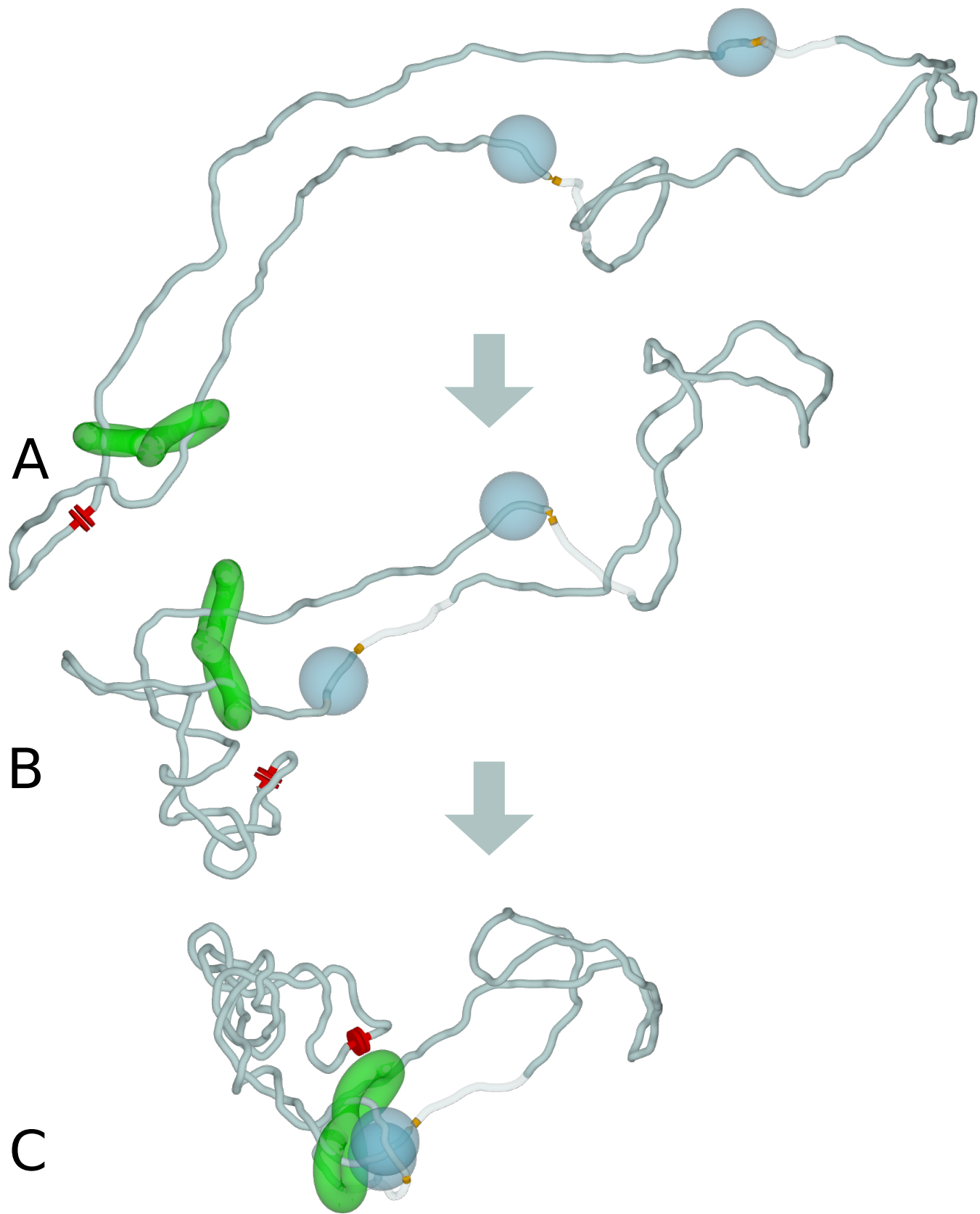

Supplement: Supplementary Data [file gkx1123_supp.zip › Figure S3.pdf]

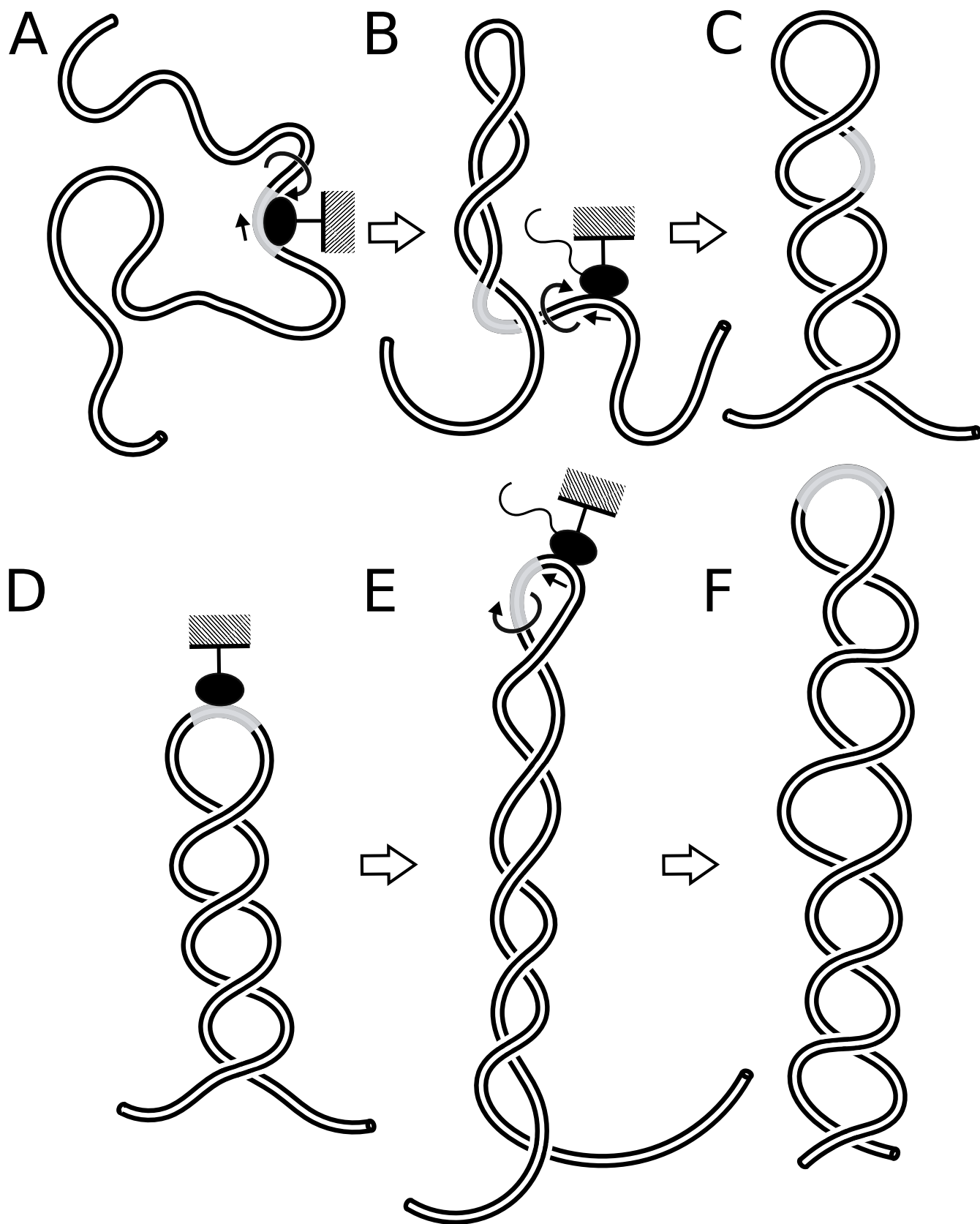

Supplement: Supplementary Data [file gkx1123_supp.zip › Figure S4.pdf]
